# Action potential broadening in a presynaptic channelopathy

**DOI:** 10.1038/ncomms12102

**Published:** 2016-07-06

**Authors:** Rahima Begum, Yamina Bakiri, Kirill E. Volynski, Dimitri M. Kullmann

**Affiliations:** 1 UCL Institute of Neurology, University College London, Queen Square, London WC1N 3BG, UK

## Abstract

Brain development and interictal function are unaffected in many paroxysmal neurological channelopathies, possibly explained by homoeostatic plasticity of synaptic transmission. Episodic ataxia type 1 is caused by missense mutations of the potassium channel Kv1.1, which is abundantly expressed in the terminals of cerebellar basket cells. Presynaptic action potentials of small inhibitory terminals have not been characterized, and it is not known whether developmental plasticity compensates for the effects of Kv1.1 dysfunction. Here we use visually targeted patch-clamp recordings from basket cell terminals of mice harbouring an ataxia-associated mutation and their wild-type littermates. Presynaptic spikes are followed by a pronounced afterdepolarization, and are broadened by pharmacological blockade of Kv1.1 or by a dominant ataxia-associated mutation. Somatic recordings fail to detect such changes. Spike broadening leads to increased Ca^2+^ influx and GABA release, and decreased spontaneous Purkinje cell firing. We find no evidence for developmental compensation for inherited Kv1.1 dysfunction.

## Abstract (web summary)

Episodic ataxia type 1 is caused by mutations in the potassium channel Kv1.1, which is found in cerebellar basket cells. Here, the authors use electrophysiology techniques to characterize these mutant channels, and observe that the changes result in decreased spontaneous Purkinje cell firing with no evidence for developmental compensation.

## Graphical abstract

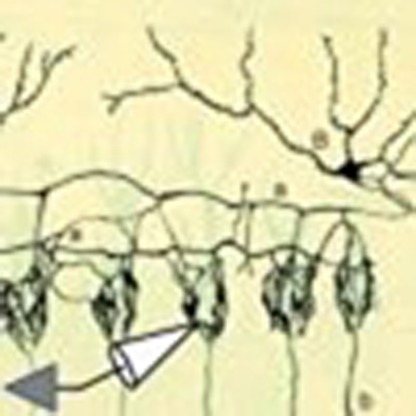

Kv1 channels are mainly expressed in axons throughout the central nervous system and play important roles in action potential initiation and repolarization^1^,^2^,^3^. Several heterozygous mutations in the *KCNA1* gene, which encodes the Kv1.1 subunit, have been identified in episodic ataxia type 1 (EA1), a familial disorder characterized by paroxysmal cerebellar incoordination and interictal myokymia, and occasionally with other features such as epilepsy^4^,^5^,^6^,^7^,^8^. Consistent with the prominent features of the syndrome, Kv1.1 is most abundantly expressed in the terminals of cerebellar basket cells^9^,^10^,^11^,^12^.

Heterologous expression studies have shown that mutations disrupt channel function through a variety of changes in assembly, trafficking and kinetics, often with dominant negative effects^13^,^14^,^15^. The consequences depend on the channel composition, as Kv1.1 co-assembles with other members of the Kv1 family and with beta subunits, and it is therefore difficult to predict the consequence of a given mutation *in situ* from heterologous expression studies. Indeed, some mutations may interfere with fast inactivation, offsetting other loss-of-function effects^16^,^17^. A mouse knock-in model of EA1 (*Kcna1*^V408A/+^) bypasses these limitations of heterologous expression, and exhibits impaired cerebellar coordination when stressed^18^. Importantly, and unlike most mouse models of human genetic disease, the disease phenotype is recapitulated in the heterozygous state^18^.

How does Kv1.1 channel dysfunction affect synaptic and circuit function, leading to cerebellar incoordination? In the neocortex, Kv1 channels play a key role in action potential repolarization^1^, and also determine the latency to the first action potential in fast-spiking interneurons^2^,^19^. Little is known, however, of the consequences of Kv1.1 mutations in the cerebellar cortex. A study using targeted patch-clamp recordings showed that α-dendrotoxin (α-DTx), a blocker of Kv1.1 and Kv1.2 channels^20^, attenuates K^+^ currents in voltage-clamp recordings from basket cell terminals, where both channel subunits are highly concentrated^21^. However, the consequences for action potential shape are unknown, and current-clamp recordings have not been reported. Although α-DTx increased spontaneous inhibitory postsynaptic currents (IPSCs) in Purkinje cells^22^, in another study it had no effect on action potential-dependent Ca^2+^ fluorescence transients measured from basket cell boutons^23^. Moreover, it is difficult to extrapolate from acute pharmacological blockade of Kv1.1 and Kv1.2 channels to constitutive dysfunction of Kv1.1 with dominant negative effects that depend on channel stoichiometry^13^,^14^,^15^,^16^,^17^. The *Kcna1*^V408A/+^ mouse shows an increase in spontaneous IPSC frequency in Purkinje cells relative to wild-type littermates^18^, but how this relates to action potential-dependent GABA release from basket cell terminals is unclear. Furthermore, abundant evidence exists for developmental homoeostatic compensation for pharmacological manipulation of ion channels and receptors^24^. Do similar compensatory mechanisms occur in response to inherited mutations of presynaptic ion channels? To address these questions, we first characterized presynaptic action potentials at cerebellar basket cell terminals of wild-type mice, and then compared the effects of pharmacological and genetic manipulations of Kv1.1 on presynaptic spike shape, action potential Ca^2+^ influx, and GABAergic inhibition of Purkinje cells.

## Results

### Presynaptic spikes are broadened by Kv1.1 blockade

**Figure 1:**
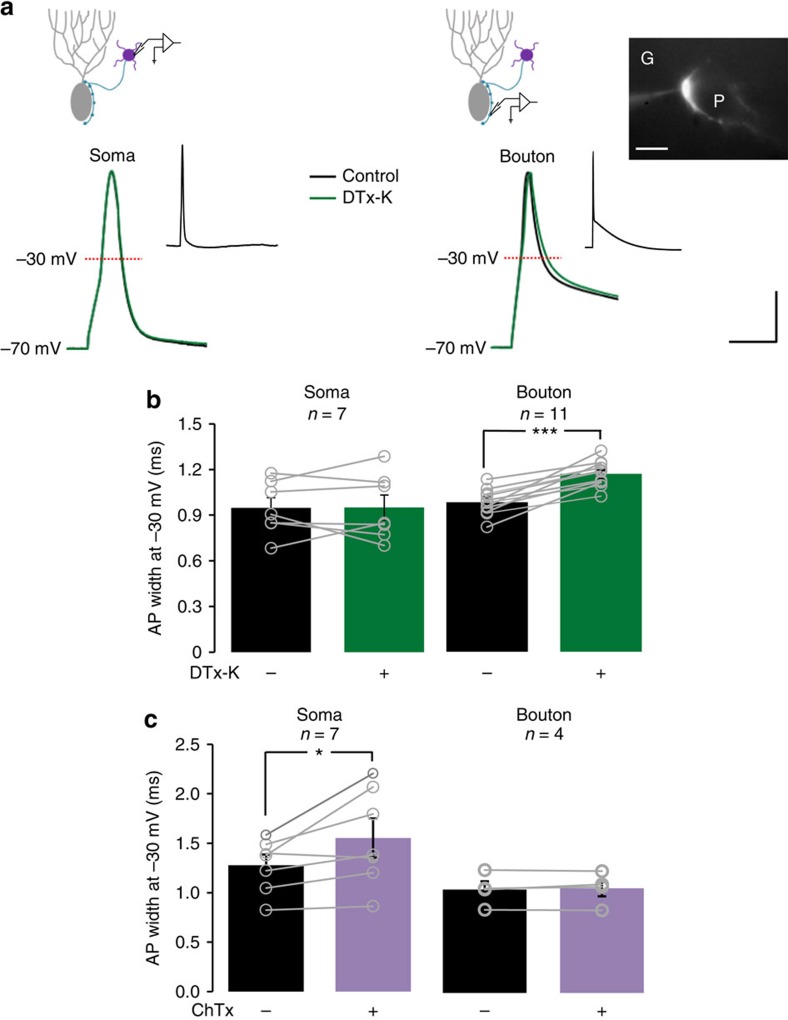
Compartment-specific roles of K^+^ channels in action potential shape in cerebellar basket cells. (**a**) Representative action potentials recorded from the soma (left) or bouton (right) of cerebellar basket cells, before (black trace) and after (green trace) Kv1.1 blockade with DTx-K. Top: schematics illustrating recording sites. Inset: action potentials at slow time base, showing a prominent ADP at the bouton. Scale bar, 20 mV; 2 ms (main traces); 40 mV, 200 ms (insets). The fluorescence image at right shows a cerebellar basket cell bouton labelled with Alexa 568. G and P indicate granule cell and Purkinje cell layer, respectively. Scale bar, 10 μm. (**b**) Summary data showing selective effect of DTx-K on presynaptic spike width measured at –30 mV. Circles show individual experiments. (**c**) BK_Ca_ blockade with ChTx led to somatic action potential broadening. *P<0.05, ***P<0.001; paired *t*-tests.

To understand the normal role of Kv1.1 in basket cells we examined the effect of the highly specific Kv1.1 blocker DTx-K^25^ on action potential shape. Targeted patch-clamp recordings were obtained from somata or terminals of basket cells in acute cerebellar slices from wild-type mice, bred on the same background as *Kcna1*^V408A/+^ mice described below^18^. Somatic recordings from basket cells showed no effect of 200 nM DTx-K on action potential width (Fig. 1a,b). A likely explanation is that Kv1.1 is predominantly expressed in axons and terminals of basket cells^9^,^11^, and plays only a small role in somata. Patch-clamp recordings from basket cells have indeed detected potassium currents with biophysical and pharmacological properties consistent with Kv1.1 or Kv1.2 (refs 21, 26). We therefore targeted basket cell terminals for patch-clamp recordings.

Presynaptic terminals were initially identified under infrared differential interference contrast (DIC), and had a high input resistance, little or no sag potential, and were able to spike upon 1-ms current injection (passive membrane properties are given in Supplementary Table 1). Prolonged depolarizing current pulses failed to evoke stable trains of action potentials (see also Supplementary Fig. 1a). Instead, repeated brief pulses reliably triggered action potentials at either 55 or 100 Hz (see also Supplementary Fig. 1b,c), with modest progressive spike broadening. Evoked presynaptic action potentials were followed by a prominent afterdepolarization (ADP), which reversed between –50 and –40 mV (see also Supplementary Fig. 2a,b). Because GABA_A_ autoreceptors are prominent in another type of cerebellar interneurons, stellate cells^27^, we asked whether the ADP was abolished by blocking these receptors. Bath application of picrotoxin (PTx, 100 μM), however, only led to a small albeit significant decrease in the area of the ADP (Supplementary Fig. 2c,d), suggesting that GABA release from basket cell terminals acting on autoreceptors plays only a small role.

In contrast to somatic action potentials, presynaptic spike width (measured at –30 mV) was robustly prolonged by DTx-K (Fig. 1a,b; baseline: 0.96±0.03 ms; DTx-K: 1.15±0.02 ms; *P*<0.001, paired *t*-test, *n*=11). The ADP was however unaffected. We compared the effects of DTx-K to those of charybdotoxin (ChTx), a blocker of the large conductance calcium- and voltage-activated potassium channel BK_Ca_, which is usually abundantly expressed at presynaptic terminals in close association with the active zone^28^ (but see ref. 29). Unexpectedly, ChTx (100 nM) led to spike broadening at the soma (Fig. 1c: baseline: 1.28±0.10 ms; ChTx: 1.55±0.13 ms; *P*<0.05, paired *t*-test, *n*=7) but not in presynaptic boutons. Thus, two K^+^ channels show complementary roles in determining spike shape in basket cells.

### Kv1.1 modulates presynaptic Ca^2+^ influx and GABA release

**Figure 2:**
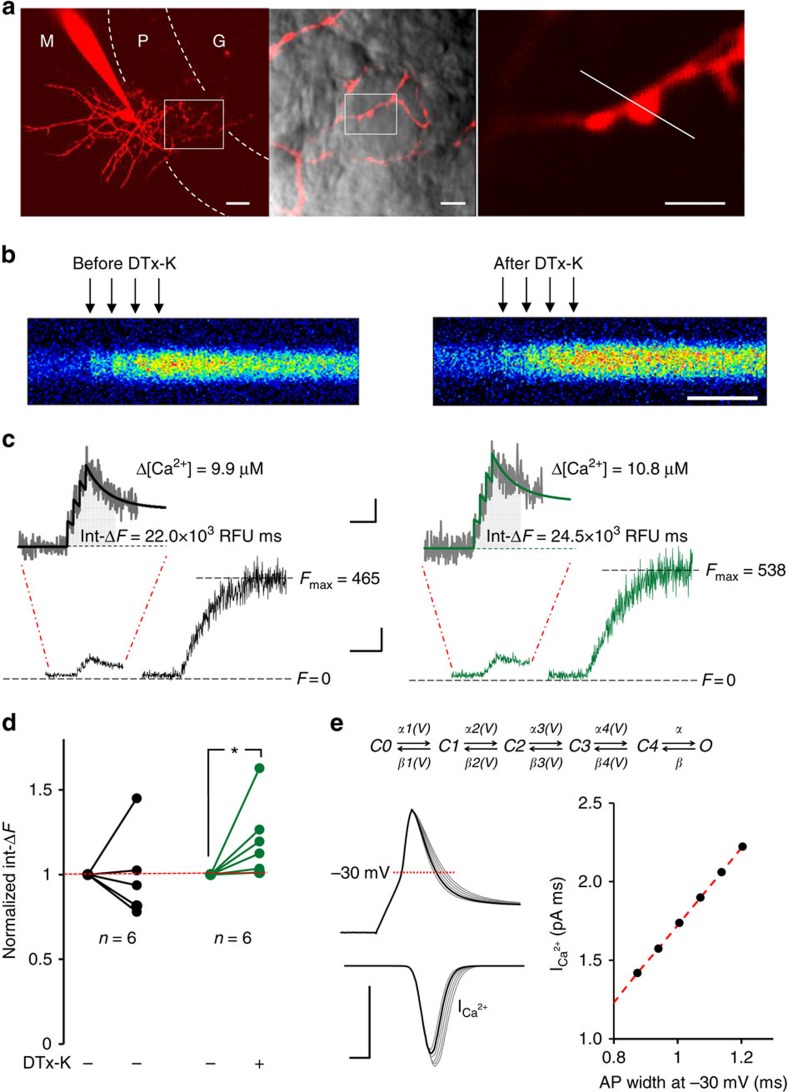
Kv1.1 modulates presynaptic action potential-evoked Ca^2+^ influx and GABA release. (**a**) Cerebellar basket cell filled with Alexa 594, under multi-photon fluorescence microscopy. M, P and G indicate molecular, Purkinje cell and granule cell layers, respectively. Middle: axon superimposed on transmitted light image, showing boutons apposed to the soma of a Purkinje cell. Inset (expanded at right) shows imaged bouton and line scan position (white dashed line). Scale bar, (left) 20 μm; (middle) 5 μm; (right) 2 μm. (**b**) Representative line scans (averages of four trials), showing Fluo-4 fluorescence response to a train of four action potentials at 40 Hz (arrows) elicited at the soma before (left) and 10 min after DTx-K perfusion (right). Scale bar, 75 ms. (**c**) Fluo-4 fluorescence time courses (same bouton as in **b**) elicited by four action potentials at 40 Hz followed by 50 action potentials at 100 Hz to saturate Fluo-4 (Scale bar, 200 relative fluorescence units (RFU); 200 ms). Insets: zoomed responses to four action potentials (Scale bar, 50 RFU; 100 ms). Black and green lines represent non-stationary single-compartment model fits before and after application of DTx-K, respectively. Model-predicted values for the total action potential-evoked Ca^2+^ influx (Δ[Ca^2+^]) are shown next to each trace. Shaded areas under the traces indicate the Fluo-4 fluorescence integrated over 200 ms from the beginning of stimulation (Int-Δ*F*), which is proportional to Δ[Ca^2+^] (Supplementary Fig. 3). (**d**) Summary of the normalized effects of DTx-K on Int-Δ*F*, compared with control experiments where DTx-K was not applied. *P<0.05, Wilcoxon signed-rank test for paired data. (**e**) Top, gating model for P/Q-type presynaptic Ca^2+^ channels^31^. Left, average time course of Ca^2+^ current through a single P/Q-type channel (10,000 simulations including failures) elicited by action potentials of different widths. Modified action potential waveforms (grey) were generated by scaling the repolarization phase of the experimental control trace (black) between 0.9 and 1.4. Right, calculated dependence of evoked Ca^2+^ current integral (I_Ca^2+^_) on action potential width measured at –30 mV.

A previous study reported no effect of α-DTx on action potential-evoked Ca^2+^ fluorescence transients measured in basket cell terminals^23^. We re-examined the role of Kv1.1 in presynaptic spike-evoked Ca^2+^ influx by patch clamping basket cell bodies in wild-type mice. Two-photon fluorescence excitation microscopy was used to image individual boutons apposed to Purkinje cell somata (Fig. 2a). Line scans were taken before and after bath perfusion of 200 nM DTx-K (Fig. 2b,c, see Methods for detailed protocols and calibration of Ca^2+^ responses). To improve the signal-to-noise ratio, we took the integral of the Fluo-4 fluorescence signal for 200 ms from the first action potential, as a measure of total action potential-evoked Ca^2+^ influx Δ[Ca^2+^]. A non-stationary single-compartment model^30^ incorporating the Ca^2+^ buffer parvalbumin provided a good fit to the fluorescence transients (Fig. 2c), confirmed that the 200 ms integral varied linearly with Δ[Ca^2+^] (see also Supplementary Fig. 3), and further yielded an estimate of the absolute Ca^2+^ concentration change.

DTx-K perfusion led to a 21±9% increase in the normalized Ca^2+^ fluorescence integral (*P*<0.05, Wilcoxon signed-rank test for paired data), which was not seen in control experiments followed for the same time (Fig. 2d). Dividing the estimated total Ca^2+^ concentration change (Δ[Ca^2+^] ∼10 μM per action potential under baseline conditions) into the approximate bouton volume (estimated from DIC or Alexa images as roughly 1 fL), we further estimated that ∼6.0 × 10^6^ Ca^2+^ ions (equivalent to a charge of 1.93 fC) enter the bouton for each action potential under baseline conditions. A six-state kinetic model of P/Q-type Ca^2+^ channels^31^, which predominate in basket cells^32^, yields an estimate of their gating kinetics when driven by a presynaptic action potential waveform that incorporates the ADP recorded in boutons. Taking into account the single channel conductance and driving force^30^, we estimate that ∼ 0.04 fC enters via each Ca^2+^ channel. This yields an estimate of ∼50 P/Q-type Ca^2+^ channels present in a typical bouton. Although the driving force for Ca^2+^ entry increases following repolarization, we found that subtracting the ADP from the action potential waveform actually led to a ∼3% decrease in the total Ca^2+^ influx (Supplementary Fig. 4), consistent with accelerated deactivation. In contrast, prolonging the decay phase of the action potential led to a linear increase in Ca^2+^ influx over a wide range (Fig. 2e). This gives an independent estimate of the effect of spike broadening due to DTx-K, corresponding to an 18% increase in Ca^2+^ influx.

Two independent approaches (Ca^2+^ fluorescence imaging and kinetic modelling of P/Q-type channels), thus converge on the conclusion that Kv1.1 channel blockade causes ∼20% more Ca^2+^ influx per action potential.

Previous studies using α-DTx revealed a large increase in spontaneous IPSC amplitude and frequency in Purkinje cells^22^,^23^. We asked how DTx-K affects evoked IPSCs by recording from Purkinje cells, while activating axons in the Purkinje cell layer. Kv1.1 blockade with DTx-K led to a 45±19% increase in pharmacologically isolated monosynaptic IPSCs (*n*=15, *P*<0.05, Wilcoxon matched pairs signed-rank test; Supplementary Fig. 5). This is consistent with a ∼20% increase in Ca^2+^ influx, assuming a Ca^2+^ current cooperativity (*m*) of ∼2 (refs 33, 34).

Thus, delayed repolarization secondary to Kv1.1 blockade leads to increased Ca^2+^ influx and enhanced neurotransmitter release. How does the acute effect of manipulating Kv1.1 channels compare to genetic disruption of Kv1.1 in EA1?

### Increased spike width and GABA release in *Kcna1* ^V408A/+^ mice

**Figure 3:**
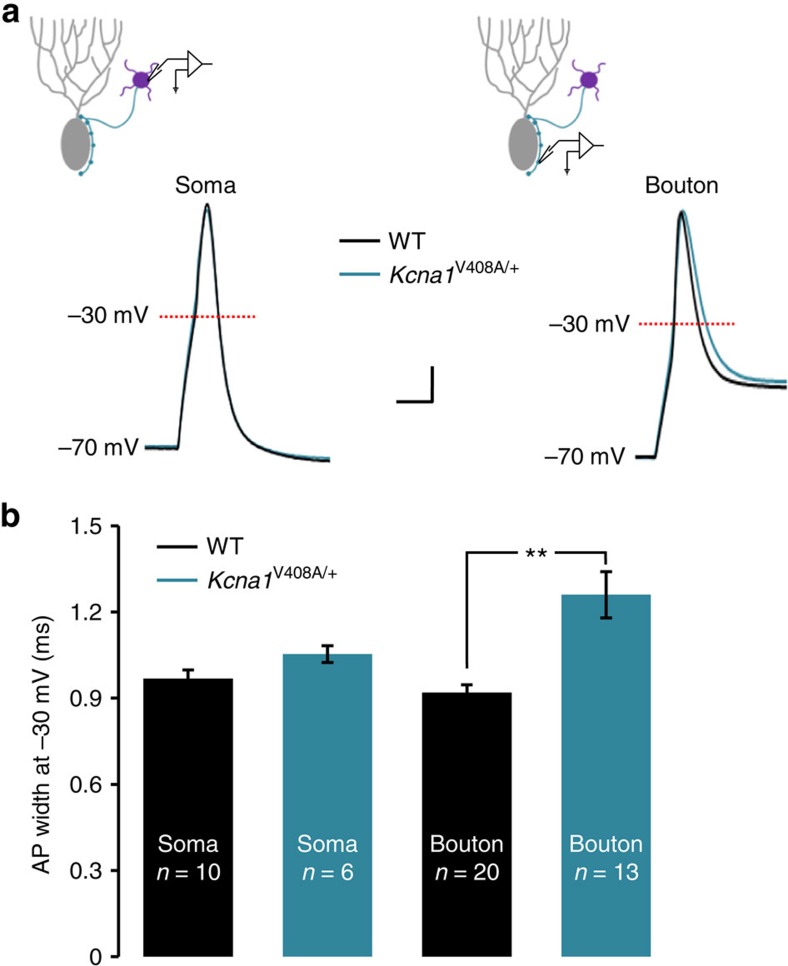
Action potentials recorded in cerebellar basket cells of *Kcna1*^V408A/+^ mice. (**a**) Superimposed representative action potentials recorded from somata (left) or boutons (right) of cerebellar basket cells from wild-type or *Kcna1*^V408A/+^ mice. Scale bar, 10 mV; 1 ms. (**b**) Presynaptic spike width at –30 mV was longer in *Kcna1*^V408A/+^ boutons. **P<0.01, unpaired *t-*test.

We repeated action potential recordings from basket cells in *Kcna1*^V408A/+^ mice, which harbour a missense mutation that underlies EA1 (ref. 18), and their wild-type littermates (*Kcna1*^+/+^). Data were acquired and analysed blind to genotype. Passive membrane properties and current threshold for eliciting action potentials were unaffected by the mutation (Supplementary Table 1). Recordings of somatic action potentials also failed to reveal a significant difference in action potential width between genotypes (Fig. 3a). A robust difference in duration was however observed in presynaptic boutons. Action potential width was ∼35% greater in *Kcna1*^V408A/+^ mice (1.26±0.08 ms, *n*=13) than in *Kcna1*^+/+^ mice (0.93±0.03 ms, *n*=20; unpaired *t*-test: *P*<0.002; Fig. 3a,b). Trains of action potentials elicited at 55 and 100 Hz showed a similar spike broadening as in wild-type mice (Supplementary Fig. 1c), showing no evidence of occlusion between the effects of repetitive spiking and genotype. DTx-K failed to broaden action potential duration recorded from the presynaptic terminals of basket cells in *Kcna1*^V408A/+^ mice, consistent with the loss of function of Kv1.1-containing channels (Supplementary Fig. 6). We also observed no difference in the ADP between the genotypes.

The *Kcna1*^V408A/+^ mutation thus broadens the spike at least as much as acute application of DTx-K in wild-type boutons. We observed no evidence of homoeostatic compensation by other channels correcting for loss of Kv1 channel function in the knock-in model of EA1.

### Increased inhibition of Purkinje cells in *Kcna1* ^V408A/+^ mice

**Figure 4:**
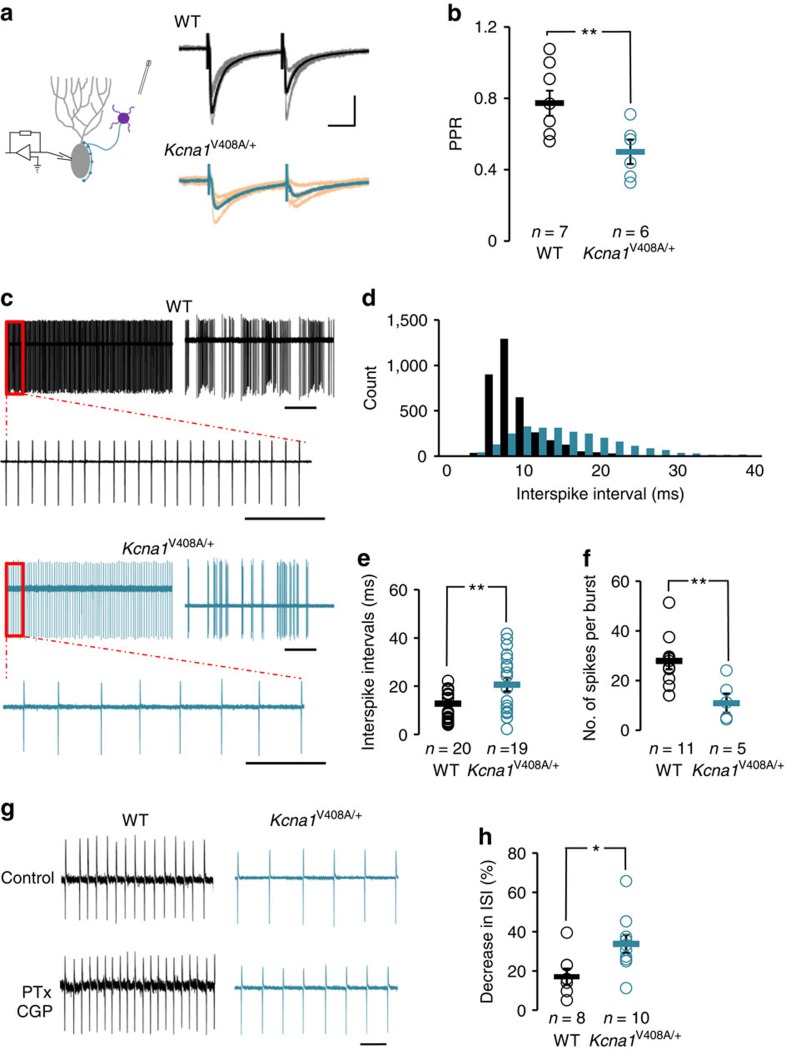
Increased GABA release at basket cell terminals in *Kcna1*^V408A/+^ mice attenuates spontaneous Purkinje cell firing. (**a**) IPSCs recorded in cerebellar Purkinje cells of wild-type and *Kcna1*^V408A/+^ mice. Grey and orange traces: individual sweeps. Black and turquoise traces: averages. Scale bar, 200 pA; 10 ms. (**b**) PPR was significantly lower in *Kcna1*^V408A/+^ than wild-type Purkinje cells. (**c**) Representative cell-attached recordings from wild-type and *Kcna1*^V408A/+^ Purkinje cells, illustrating tonic and burst firing, and periods of quiescence. Scale bar, 500 ms; 50 ms (expanded traces). (**d**) Inter-spike interval distribution during tonic firing (same cells as in **c**). (**e**) Average inter-spike interval was significantly longer in *Kcna1*^V408A/+^ than wild-type Purkinje cells. (**f**) Average number of action potentials per burst was significantly higher in wild-type neurons. (**g**) Representative traces showing a greater decrease in inter-spike interval following PTx (100 μM) and CGP (1 μM) perfusion in *Kcna1*^V408A/+^ than wild-type neurons (Scale bar, 20 ms). (**h**) Summary of effect of GABA receptor blockade. *P<0.05; **P<0.01, unpaired *t*-tests (**b**,**f**,**h**) or Mann–Whitney *U* test (**e**).

An increase in spontaneous GABAergic IPSCs has previously been reported in Purkinje cells of the *Kcna1*^V408A/+^ mouse^18^. It is technically difficult to compare presynaptic Ca^2+^ influx between genotypes, and the amplitudes of evoked IPSCs are uninformative when evoked by extracellular stimulation of multiple axons. Instead, we compared the paired-pulse ratio of IPSCs recorded in Purkinje cells as a surrogate measure of action potential-evoked neurotransmitter release probability. Paired-pulse ratio was significantly lower in *Kcna1*^V408A/+^ mice (0.49±0.06, *n*=6) than in *Kcna1*^+/+^ mice (0.77±0.07, *n*=7; *P*<0.01, unpaired *t-*test; Fig. 4a,b), consistent with an increased release probability. DTx-K had no significant effect on IPSC amplitude in *Kcna1*^V408A/+^ mice (4±5%, *n*=5). To examine the downstream consequences for Purkinje cells, we compared their spontaneous activity between *Kcna1*^V408A/+^ and *Kcna1*^+/+^ littermates, using cell-attached recordings to minimize disruption of intrinsic excitability. All Purkinje cells irrespective of genotype exhibited periods of tonic firing alternating with periods of quiescence or burst firing (Fig. 4c), as previously reported in wild-type mice and rats^35^. Overall, spike frequency recorded in *Kcna1*^V408A/+^ mice was significantly lower (55.4±10.7 Hz, *n*=13) compared with *Kcna1*^+/+^ littermates (96.3±12.1 Hz, *n*=20, *P*<0.02, Mann–Whitney *U* test). The average inter-spike interval during tonic firing was significantly longer in *Kcna1*^V408A/+^ mice (20.6±2.8 ms, *n*=19) than in *Kcna1*^+/+^ mice (10.9±1.3 ms, *n*=20; *P*<0.01, Mann–Whitney *U* test; Fig. 4d,e). The number of spikes per burst was also lower in mutant mice (10.9±3.3, *n*=5) than in wild-type mice (27.9±3.0, *n*=11; *P*<0.01, unpaired *t*-test; Fig. 4f). No significant difference was observed in burst duration or the inter-burst interval between the genotypes. There was also no difference in action potential width in Purkinje cells between the genotypes, as estimated by integrating the cell-attached recordings^36^ (Supplementary Fig. 7).

Finally, we asked if enhanced spontaneous GABA release contributed to the lower activity of Purkinje cells. Bath application of blockers of GABA_A_ and GABA_B_ receptors (100 μM PTx and 1 μM CGP 52432, respectively) led to a greater decrease in inter-spike interval in *Kcna1*^V408A/+^ mice (33.7±4.6%, *n*=10) than in *Kcna1*^+/+^ mice (17.1±4.1%, *n*=8; *P*<0.05, unpaired *t*-test; Fig. 4g,h). Indeed, the inter-spike interval in the presence of GABA receptor blockers was not significantly different between wild-type and *Kcna1*^V408A/+^ mice (11.3±1.9 ms and 15.6±2.1 ms; *P*=0.36, unpaired *t*-test). We thus conclude that enhanced GABA release indeed contributes to decreased spontaneous activity of Purkinje cells, with no evidence for a homoeostatic compensation in the EA1 mouse model.

## Discussion

The present study reveals a major role of Kv1.1 channels in action potential repolarization at basket cell terminals, which was not apparent when recording from somata. We provide a quantitative account of presynaptic spike-evoked Ca^2+^ transients. EA1 modelled in heterozygous knock-in mice led to a similar effect on spike shape and neurotransmitter release, as acute pharmacological blockade of Kv1.1 channels in wild-type mice. GABAergic inhibition of Purkinje cell firing was enhanced in *Kcna1*^V408A/+^ mice, with no evidence of homoeostatic compensation for this presynaptic channelopathy.

Central to this study is the ability to target small GABAergic presynaptic terminals for whole-cell patch-clamp recordings. This has only previously been achieved in voltage-clamp mode^21^. Presynaptic recordings have yielded invaluable insights into mechanisms linking action potentials and K^+^ channels to neurotransmitter release, but hitherto such studies have been restricted to giant calyceal synapses in the auditory brainstem^37^ or large hippocampal or cerebellar mossy fibre boutons^38^,^39^. Small boutons can also be targeted for whole-cell patch-clamp using scanning ion conductance microscopy, but this is restricted to cultured neurons^40^. Less invasive methods include bouton-attached recordings^41^ and voltage-sensitive dye imaging. A study using both of these methods in cerebellar stellate cells revealed an important role of Kv1 channels in action potential shape and activity-dependent broadening at the axon initial segment^42^. However, in striking contrast to the present results, Kv1 family channels were found to play only a minor role in boutons. The difference is consistent with the intense staining for Kv1.1 and Kv1.2, specifically in the terminals of basket cells^9^,^10^,^11^,^12^. Several other roles have been inferred for axonal Kv1.1 channels on the basis of somatic recordings. These include delayed spiking in some forebrain interneurons^2^,^19^ and subthreshold modulation of neurotransmitter release^43^.

The direct recordings from basket cell boutons reported here revealed an increase in spike width by acute application of the Kv1.1-specific blocker DTx-K, which was qualitatively similar to the effect of the *Kcna1*^V408A/+^ mutation. The *Kcna1*^V408A/+^ mutation, however, had a larger effect than pharmacological blockade (spike width at –30 mV: 1.26±0.08 ms and 1.15±0.02 ms, respectively), consistent with a dominant negative effect and heteromultimeric assembly of Kv1 channels. Mutant mice also exhibit an increase in spontaneous IPSCs, but no difference in basket cell firing rate or in miniature IPSC amplitude^18^. We failed to observe a compensation for the genetic lesion at the level of spike shape, GABA release or downstream effects on Purkinje cell firing. This is arguably unexpected, given the abundant evidence that blocking neurotransmitter receptors or Na^+^ channels for a few days results in extensive synaptic plasticity, contributing to homoeostatic regulation of neuronal activity^24^. Most attention has hitherto been given to homoeostatic plasticity at glutamatergic synapses. However, GABAergic synaptic strength has also been shown to undergo activity-dependent plasticity that compensates for altered activity^44^,^45^,^46^. The results of the present study question the importance of these phenomena for an inherited presynaptic K^+^ channelopathy. A possible explanation is that basket cell synapses are intrinsically less plastic than synapses in the forebrain, although the finding that spontaneous Purkinje cell firing was depressed in *Kcna1*^V408A/+^ mice further argues against a major role of homoeostatic plasticity in the cerebellar cortex.

The present study stresses the role of Kv1.1 in ensuring rapid repolarization. It is tempting to speculate that the very high density of Kv1.1 channels in basket cell terminals is an evolutionary adaptation related to the electrotonic properties of a relatively short but profusely arborizing axon, compromising the ability of passive charge dissipation to terminate action potentials. Basket cell terminals also have a prominent ADP that is not affected by either pharmacological or genetic manipulation of Kv1.1, or GABA_A_ receptor blockade, and whose biophysical basis remains to be elucidated. Another anatomical peculiarity of basket cell terminals, the ‘pinceau' formation, where ephaptic transmission also contributes to inhibition of Purkinje cells^47^,^48^, only forms at a later developmental stage^49^, and so is unlikely to contribute to spike shape in the present study.

We observed a complementary compartmentalization of BK_Ca_ channels, blockade of which affected somatic, but not presynaptic spike width. A pronounced effect of ChTx on somatic K^+^ currents has not been reported previously. Although BK_Ca_ channels are usually abundantly expressed presynaptically, a detailed immunhistochemical study identified cerebellar basket cell synapses as the sole exception to this rule, with greater post- than presynaptic expression^29^. The effect of BK_Ca_ channel blockade on somatic action potential width is also unexpected because they generally require a large depolarization and a high local Ca^2+^ concentration to open^50^. Although BK_Ca_ channels are present at cerebellar mossy fibre terminals, their role in action potential repolarization is masked by recruitment of fast Kv3 channels^51^. We cannot exclude a possible role for presynaptic BK_Ca_ channels in basket cells under conditions when other K^+^ channels are non-functional.

Acute blockade of Kv1.1 channels with DTx-K led to an increase in action potential-evoked Ca^2+^ influx, which was not observed in a previous study^23^. This is consistent with spike width as a major determinant of Ca^2+^ channel activation and total Ca^2+^ influx^52^. Indeed, the simulations suggest that the ADP, by comparison, has little effect on Ca^2+^ influx. We did not attempt to compare presynaptic Ca^2+^ influx in wild-type and *Kcna1*^V408A/+^ mice because of the large variability among boutons. Nevertheless, both pharmacological and genetic manipulations of Kv1.1 led to a marked increase in evoked and spontaneous GABA release, consistent with a supralinear relationship between Ca^2+^ influx and exocytosis^33^,^34^. In common with many other neurological channelopathies, the paroxysmal nature of EA1 remains unexplained. Although we have only examined in detail one synapse in the cerebellar circuit, where Kv1.1 is most abundantly expressed, we cannot exclude a role for other elements of the cerebellar circuitry. Strikingly, cerebellar incoordination both in patients with EA1 and in mice harbouring the V408A mutation is triggered by stress, suggesting a role for neuromodulators.

## Methods

### Cerebellar slices

*Kcna1*^V408A/+^ mice were a generous gift from J. Maylie. Breeding pairs were set-up between a heterozygous *Kcna1*^V408A/+^ female and a wild-type C57/BL6 mouse (Harlan), and refreshed after 6 months. This study was performed in accordance with the Animals (Scientific Procedures) Act 1986.

All experiments were performed and analysed blind to genotype. Acute parasagittal cerebellar slices (300-μm thick) were prepared from postnatal day 14–20 mutant and wild-type littermates using a vibrating microtome (Leica VT 1200) in an ice-cold solution containing (in mM): 75 sucrose, 87 NaCl, 2.5 KCl, 25 NaHCO_3_, 1.25 NaH_2_PO_4_, 7 MgCl_2_, 0.5 CaCl_2_ and 20 glucose, (pH=7.4 when gassed with 95% O_2_:5% CO_2_). Slices were warmed to 32 °C for 10 min and then stored in a carbogen-gassed solution at room temperature containing (in mM): 126 NaCl, 2.5 KCl, 25 NaHCO_3_, 1.25 NaH_2_PO_4_, 1 MgCl_2_, 2.0 CaCl_2_ and 10 glucose. The same solution was used to perfuse slices during electrophysiological recordings. Slices were used within 3 h of preparation.

### Electrophysiology

Slices were anchored in a recording chamber mounted on the stage of an upright microscope (BX51WI, Olympus) and visualized with infrared differential interference contrast optics with a × 20 water immersion objective. Slices were continuously perfused at a rate of 3 ml min^−1^ and recordings were obtained at 33–35 °C. AMPA and NMDA receptors were blocked throughout. Purkinje cells and basket cell somata were patch clamped with 4–6-MΩ glass pipettes, to achieve an access resistance <20 MΩ. Bouton recordings were obtained with 9–11-MΩ glass pipettes, to achieve an access resistance of <50 MΩ. Basket cell boutons were identified by their size, shape, apposition to Purkinje cell somata and input resistance ∼1 GΩ.

For current-clamp experiments the pipette solution contained (in mM): 134 K-gluconate; 5 KCl, 10 phosphocreatine, 10 HEPES, 2–5 EGTA, 0.3 Na_3_GTP and 4 MgATP, as well as 2 mg ml^−1^ biocytin or 200 nM Alexa 568 (adjusted to pH 7.2–7.3 with KOH). If necessary, current was injected to maintain the membrane potential between –70 and –75 mV. Boutons could be made to fire one-to-one in response to 0.5- or 1-ms current injections delivered at 55 or 100 Hz.

For voltage-clamp experiments, the pipette solution contained (in mM): 140 CsCl; 1 EGTA, 10 HEPES, 1 MgCl_2_, 0.3 Na_3_GTP, 5 MgATP, 1–2 QX314-Br and pH 7.2–7.3 with CsOH. Purkinje cell somata were voltage clamped at –70 mV. Whole-cell capacitance and series resistance were compensated. IPSCs were elicited in Purkinje cells using an extracellular stimulating electrode placed in the molecular layer.

To measure the spontaneous firing rates of Purkinje cells, pipettes were filled with the perfusion solution, and voltage-clamp recordings were obtained in the cell-attached mode.

Data were acquired using a Multiclamp 700B amplifier (Molecular Devices) and custom software (National Instruments LabView). Data were low-pass filtered (20 kHz) and digitized at 50 kHz.

All data from *Kcna1*^V408A/+^ mice were compared with data from their wild-type littermates. Data were analysed using LabView and are presented as mean±s.e.m. The action potential width was measured at –30 mV. Recordings where action potentials did not overshoot 0 mV were discarded. To calculate the area under the ADP curves the initial 20 ms were used. In graphs, open circles represent individual experiments, bars illustrate the averages obtained from all experiments. The paired-pulse ratio was calculated as the ratio of the second IPSC to the second IPSC evoked by two extracellular stimuli at 40 Hz. The voltage threshold was estimated from the maximum of the second time derivative of the voltage.

### Two-photon excitation fluorescence imaging

Basket cells held in current-clamp whole-cell mode were loaded with both a morphological dye (Alexa 594, 50 or 100 μM) and the Ca^2+^ indicator Fluo-4 (200 μM), and imaged in two-photon excitation mode at 800 nm using a femtosecond Ti:sapphire-pulsed laser (MaiTai, Spectra-Physics). Recordings started at least 20 min after obtaining the whole-cell configuration. We followed the axon and focused on presynaptic boutons in the Purkinje cell layer. Four 1-ms long depolarizing current pulses were injected at 40 Hz to evoke action potentials, and the Fluo-4 fluorescence was measured using line scans across the bouton width. Three or four trials were recorded with an inter-trial interval between 30 s and 1 min, and the saturated fluorescence (*F*_max_) was measured with a train of 50 action potentials at 100 Hz. The sequence was repeated at least 10 min after washing in DTx (200 nM). To improve the signal-to-noise ratio, we took the integral of the Fluo-4 fluorescence signal for 200 ms from the first action potential (Int-Δ*F*), as a measure of total action potential-evoked Ca^2+^ influx Δ[Ca^2+^].

### Presynaptic Ca^2+^ kinetics

The total magnitude of action potential-evoked presynaptic Ca^2+^ influx Δ[Ca^2+^] was estimated using a non-stationary single-compartment model^30^,^53^,^54^. We assumed that, in addition to Fluo-4, the presynaptic bouton contained 150 μM of total parvalbumin (equivalent to 8 μM of free buffer at rest, where [Ca^2+^]_rest_=10 nM and [Mg^2+^]_rest_=0.5 mM). The model operated with only two adjustable (free) parameters: Δ[Ca^2+^] and the Ca^2+^ removal rate, which have virtually independent effects on the Ca^2+^ fluorescence signal^54^, and thus were constrained by a direct fitting procedure to match the simulated and experimental fluorescence profiles. Other model parameters were constrained by measuring the maximal Fluo-4 fluorescence, which was used to estimate [Ca^2+^]_rest_ and also the total Fluo-4 concentration at the time of each measurement. This was necessary because the Ca^2+^ indicator and morphological tracer did not fully reach a steady state during the time course of the experiment (that is, between 20 and 50 min after obtaining the whole-cell configuration; ref. 53). Ca^2+^ and Mg^2+^ binding/unbinding rates of parvalbumin were as in ref. 55, and Ca^2+^ binding/unbinding rates of Fluo-4 were as in refs 30, 53, 54.

### Ca^2+^ current modelling

Action potential-evoked Ca^2+^ currents through P/Q-type Ca^2+^ channels were modelled in the NEURON simulation environment^56^, using a six-state Ca^2+^ channel model developed for hippocampal mossy fibre boutons^31^ as described in detail previously (ref. 30).

### Drugs

To block AMPA, NMDA, GABA_A_ and GABA_B_ receptors, 5 μM 2,3-dihydroxy-6-nitro-7-sulfamoyl-benzo[f]quinoxaline-2,3-dione, 50 μM (2 *R*)-amino-5-phosphonovaleric acid (APV), 100 μM PTx and 1 μM CGP 52432, were added to the perfusate. DTx-K (200 nM) was used to block Kv1.1 channels. ChTx (100 μM) was used to block BK_Ca_ channels. APV was from Ascent (UK). DTx-K was from Alomone lab (UK). All other compounds were purchased from Tocris (Bristol).

### Statistics

Data sets that passed the Shapiro–Wilk test for normality were analysed with Student's paired or unpaired *t*-test. Non-parametric tests (Wilcoxon matched pairs signed-rank or Mann–Whitney U) were applied in all other cases.

### Data availability

The data that support the findings of this study are available from the corresponding author upon request.

## Additional information

**How to cite this article:** Begum, R. *et al.* Action potential broadening in a presynaptic channelopathy. *Nat. Commun.* 7:12102 doi: 10.1038/ncomms12102 (2016).

## Supplementary Material

Supplementary Information Supplementary Figures 1-7 and Supplementary Table 1.
