# Supplementary material for: Action potential broadening in a presynaptic channelopathy
Source: Nat Commun. 2016 Jul 6;7:12102. doi: 10.1038/ncomms12102 (PMC4935806; doi:10.1038/ncomms12102)
Supplement: Supplementary Information — Supplementary Figures 1-7 and Supplementary Table 1. [file ncomms12102-s1.pdf]

Supplementary Fig. 1

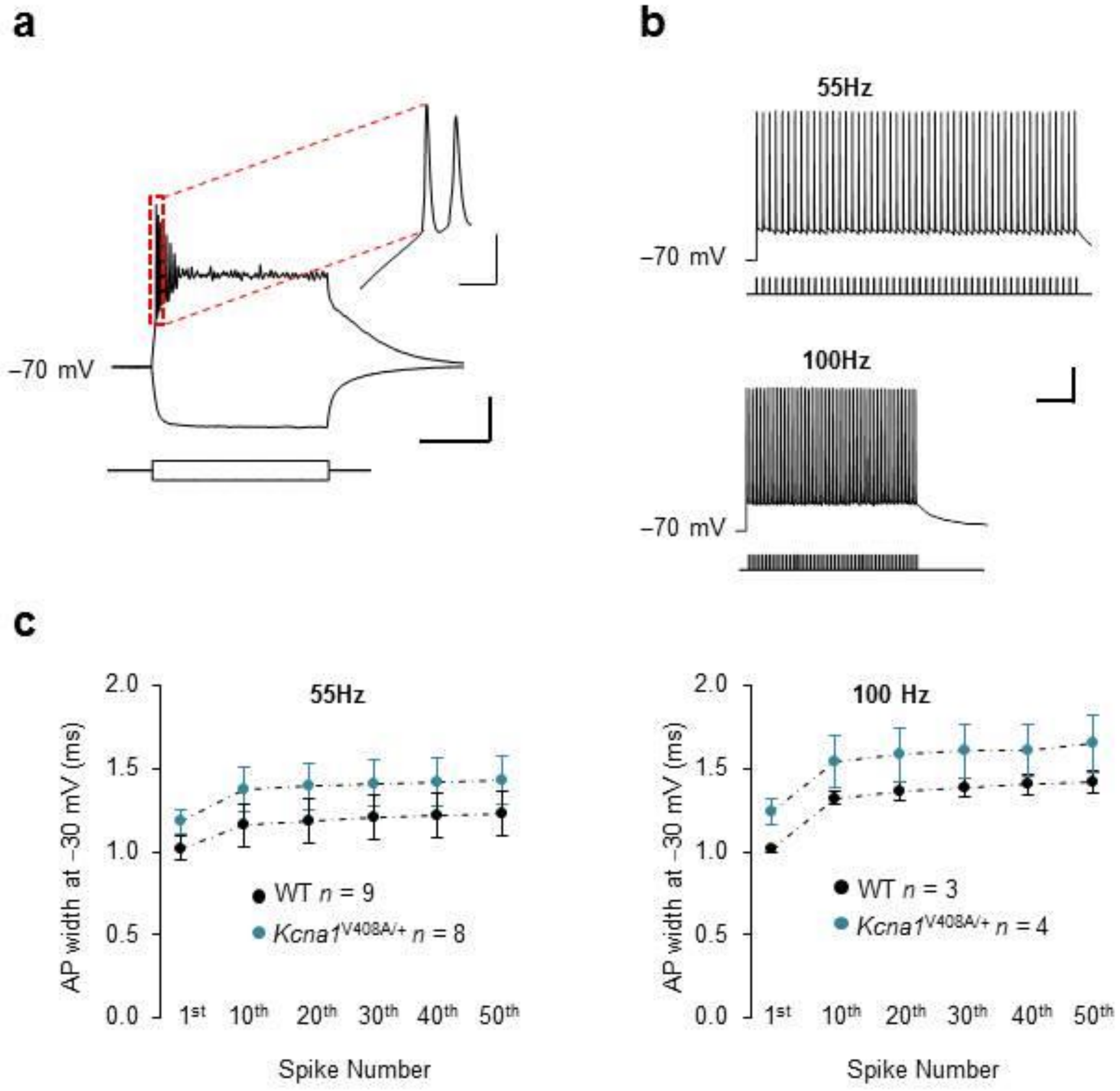

Supplementary Fig. 1: Properties of basket cell terminals.
(**a**) Prolonged depolarizing currents failed to elicit repetitive firing. Representative traces from one bouton in response to 500 ms depolarizing and hyperpolarizing 40 pA current pulses. Scale: 20 mV, 200 ms (main traces), 20mV, 5 ms (insets). (**b**) Action potentials evoked by 55 Hz and 100 Hz trains of 1 ms depolarizing pulses, scale: 20 mV, 100 ms. (**c**) Action potential broadening at 55 Hz or 100 Hz in wild type and *Kcna1*$^{V408A/+}$ mice.

Supplementary Fig. 2

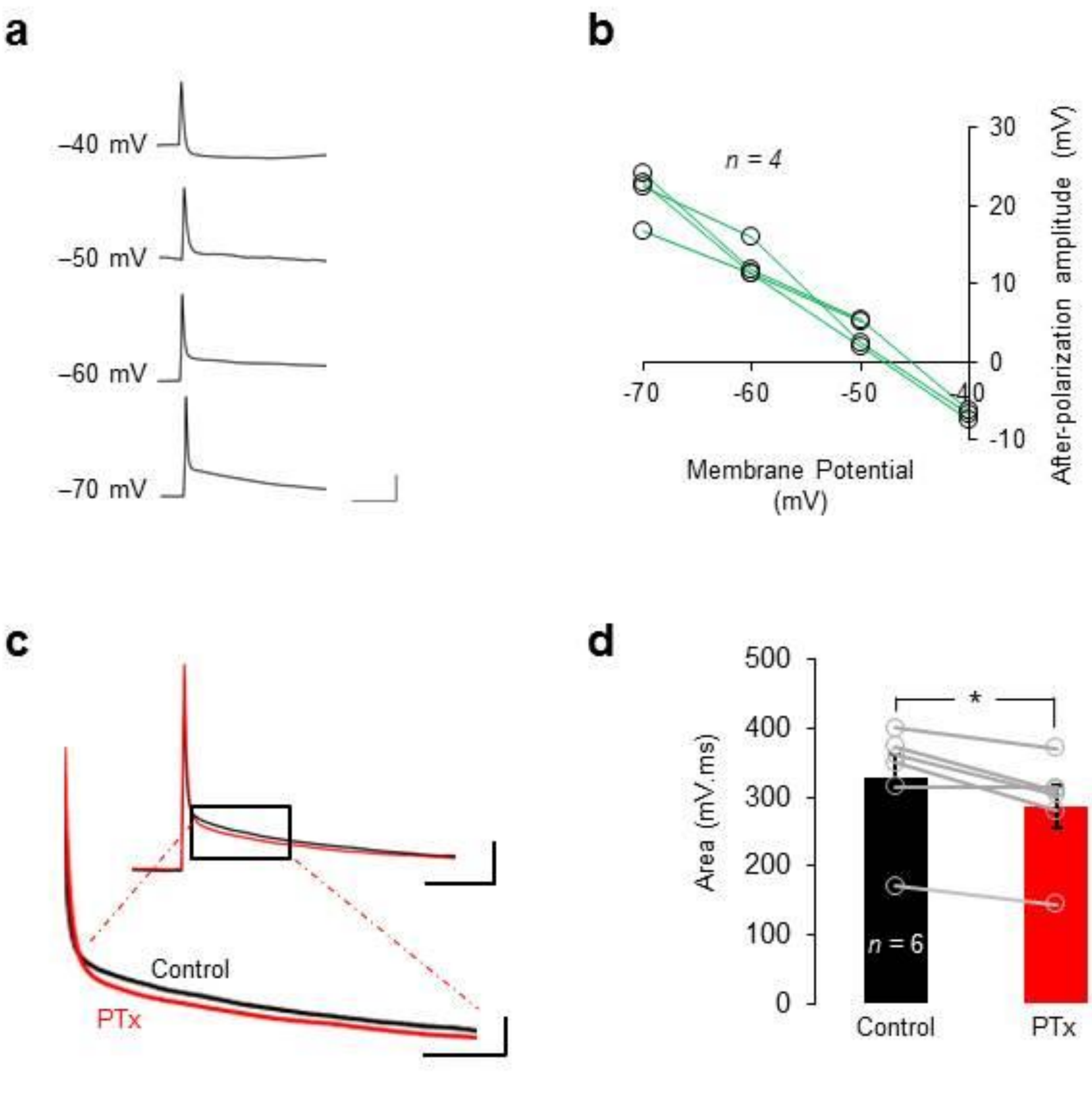

Supplementary Fig. 2. Bouton ADP properties.
(**a**) Representative traces from one bouton showing reversal of the ADP around –48 mV. Scale: 20 mV, 20 ms. (**b**) Summary data. (**c**) Representative traces from one bouton showing the effect of $GABA_A$ receptor blockade with picrotoxin (PTx). Scale: 20 mV, 20 ms (main traces); 5 mV, 5 ms (expanded trace). (**d**) PTx only decreased the ADP area measured over the initial 20 ms by a small amount (baseline: 329 ± 34 mV.ms; PTx: 287 ± 31 mV.ms). * p <0.05, paired t test, n = 6.

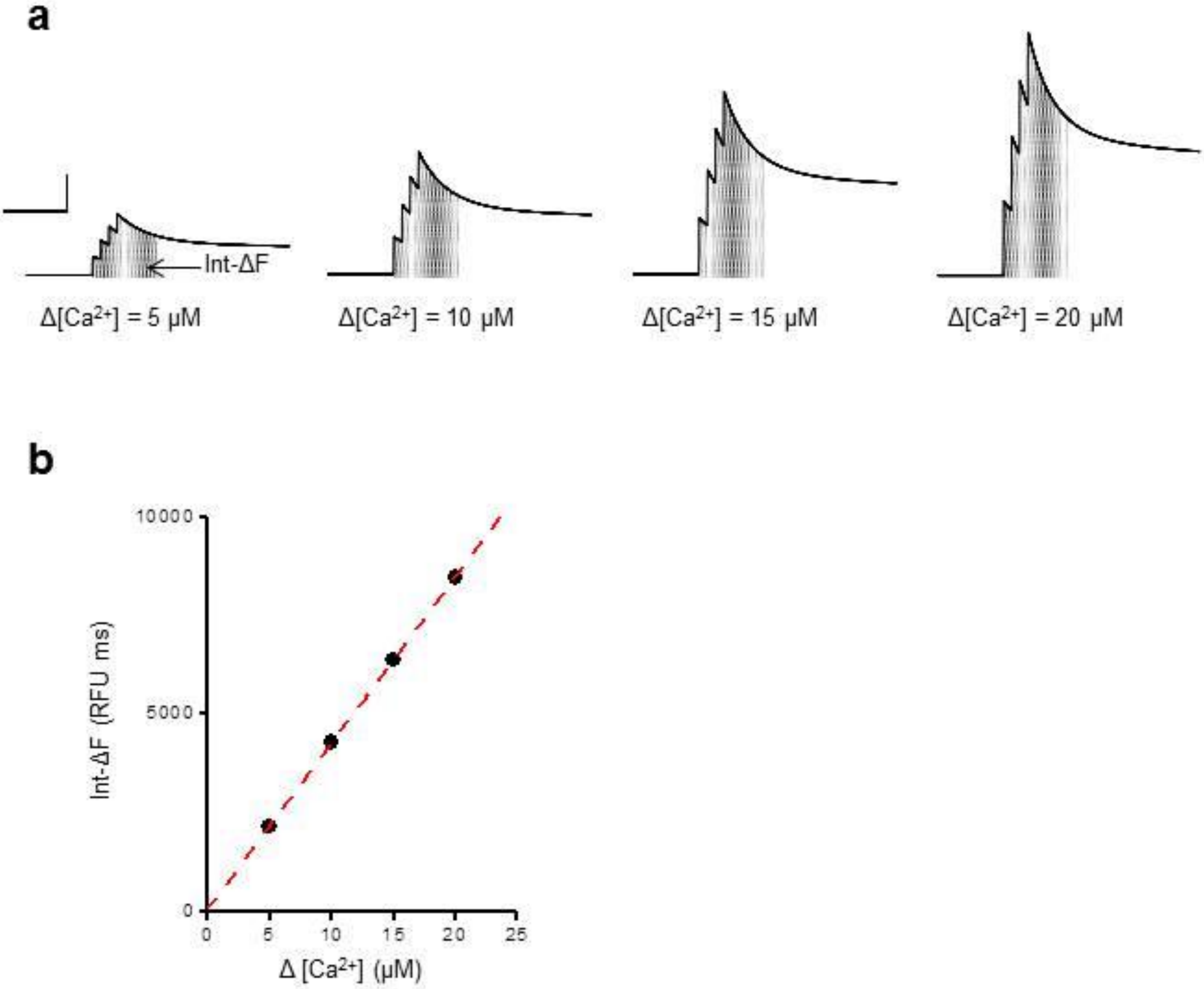

Supplementary Fig. 3. Fluo-4 fluorescence integrated over 200 ms (Int-ΔF) is proportional to total action potential-evoked Δ[Ca^{2+}].

(**a**) Fluo-4 Ca^{2+} fluorescence responses to a 40 Hz burst of 4 action potentials, simulated using a non-stationary single compartment model of presynaptic Ca^{2+} dynamics for different values of Δ[Ca^{2+}] per action potential. Shaded area under the traces corresponds to the integral of Fluo-4 fluorescence over 200 ms, starting with the beginning of stimulation (Int-ΔF). This was used as a measure of action potential-evoked Δ[Ca^{2+}]. Scale: 10 RFU, 200 ms. (**b**) Linear relationship between Int-ΔF and Δ[Ca^{2+}].

Supplementary Fig. 4

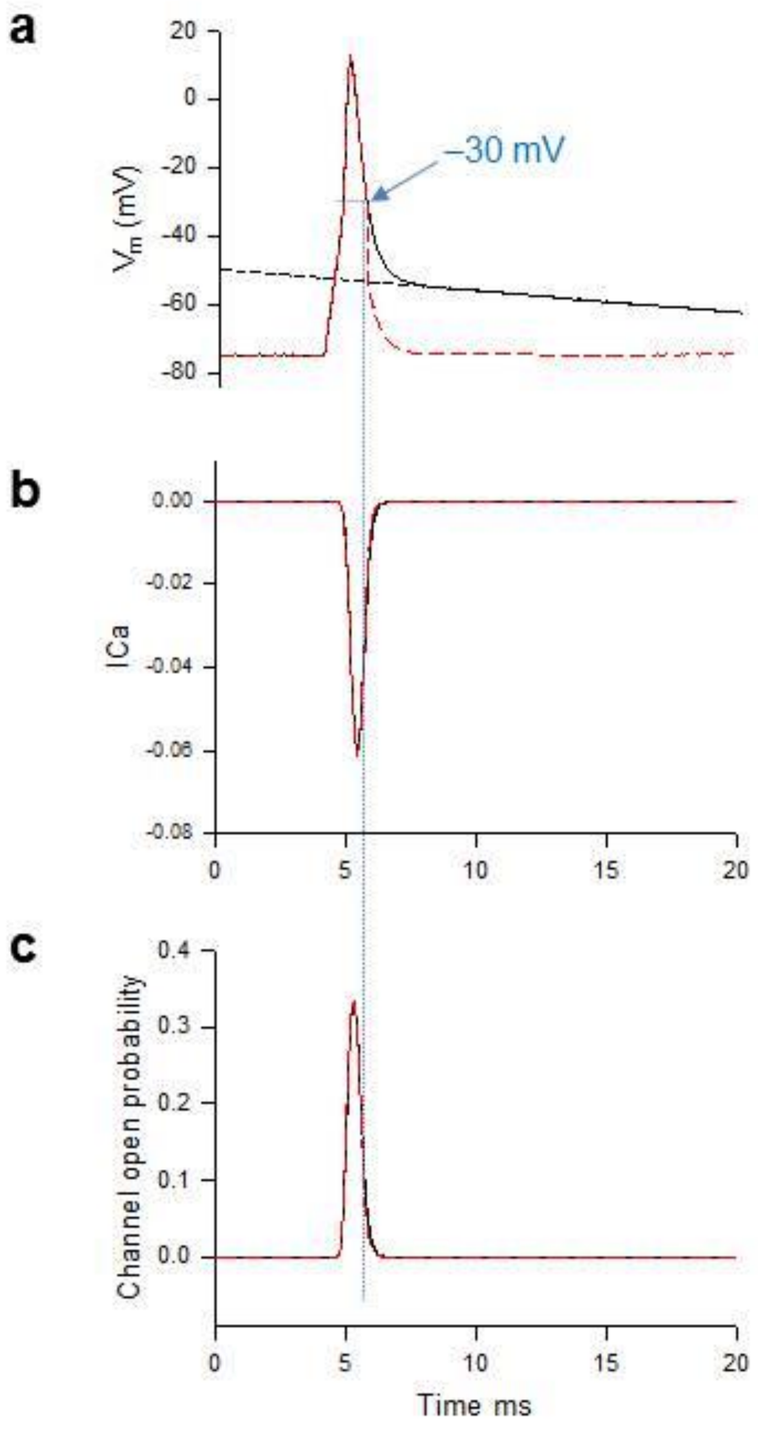

Supplementary Fig. 4. The ADP has a small effect on Ca^{2+} entry.
(**a**) The ADP was modelled by fitting a straight line to the slow voltage relaxation following a representative presynaptic action potential (dashed black line). It was then subtracted from the action potential waveform, starting when the membrane potential passed the –30 mV point (dashed red line). (**b**) Simulated Ca^{2+} current using six-state model (black: control; dashed red: without ADP). (**c**) Estimated open probability. Subtracting the ADP decreased the total integrated Ca^{2+} current by ~3%.

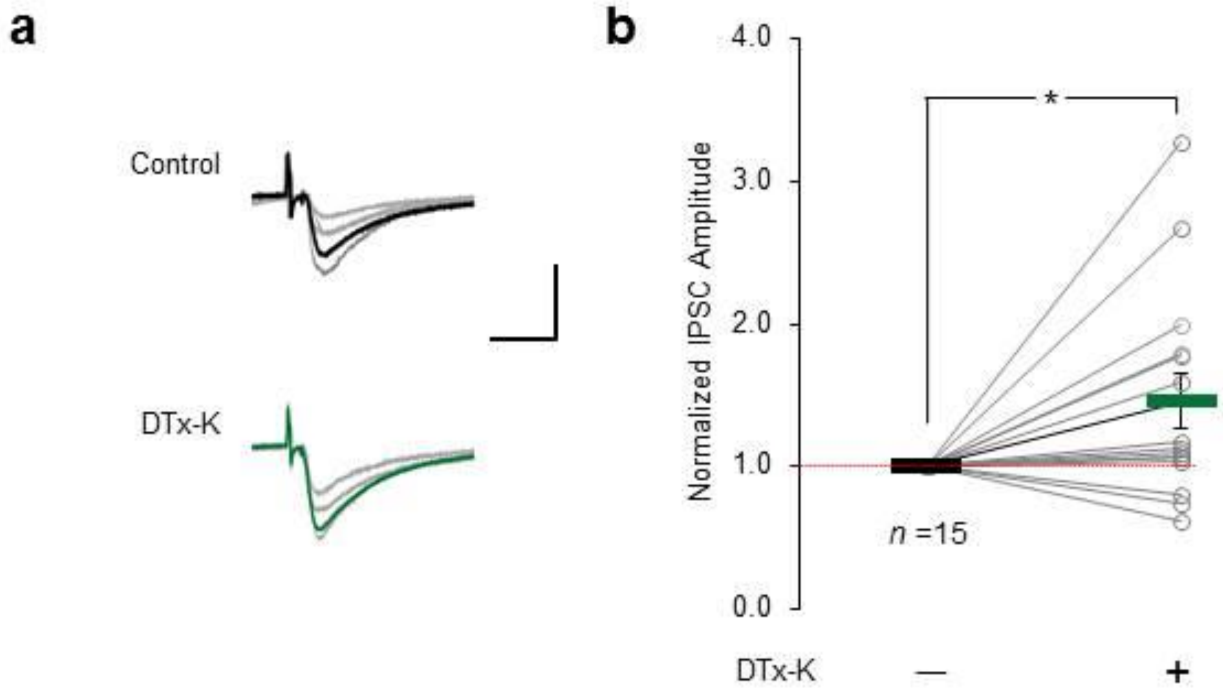

Supplementary Fig. 5. Effect of DTx-K on IPSCs recorded in wild type cerebellar Purkinje cells.

(**a**) Representative data from one experiment. Grey traces are individual sweeps; black and green traces are averages. Scale: 200 pA, 5 ms. (**b**) Summary of data. * $p < 0.05$, paired *t* test.

Supplementary Fig. 6

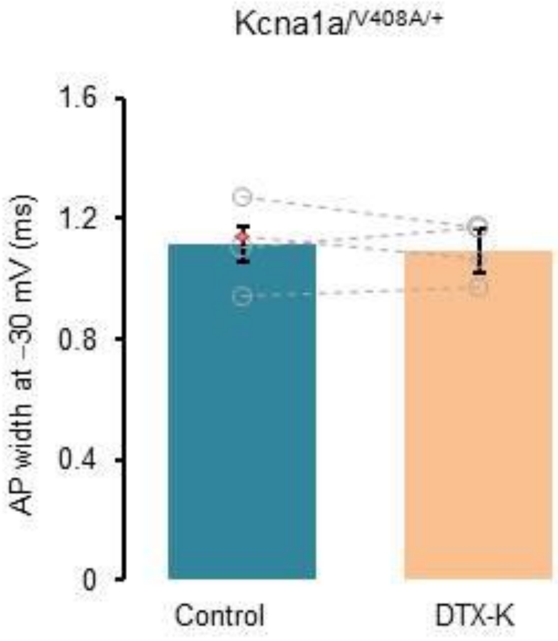

Supplementary Fig. 6. No effect of DTx-K on presynaptic spike width in *Kcna1*$^{V408A/+}$ mice.
Summary of spike widths before and after DTx-K.

Supplementary Fig. 7

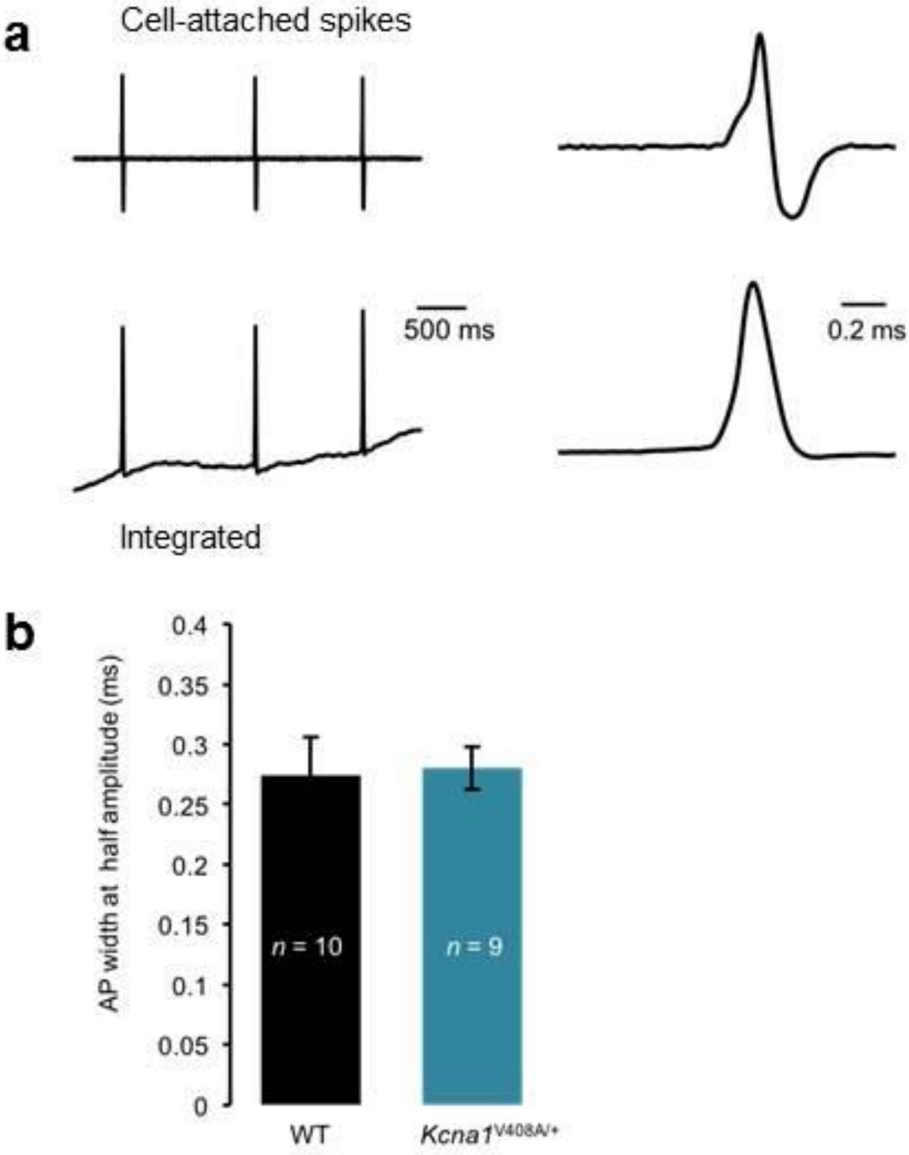

Supplementary Fig. 7. No difference in Purkinje cell spike width in *Kcna1*$^{V408A/+}$ mice. (**a**) Sample trace from one experiment showing cell-attached recording (top) and after numerical integration to estimate the spike width (bottom). Right: expanded time-base. (**b**) Comparison of wild type and *Kcna1*$^{V408A/A}$ neurons. Between 15 and 61 spikes were measured in each Purkinje cell.

Supplementary Table 1: Passive membrane properties and action potential threshold.

	WT	*Kcna1*$^{V408A/+}$
RMP	–68.8 ± 1.5 mV (n = 9)	–68.6 ± 2.3 mV (n =10)
Spike threshold	–43.2 ± 1.1 (n = 13)	–41.0 ± 0.9 (n = 21)
Rheobase	15.7 ± 1.2 pA (n = 7)	10.8 ± 4.7 pA (n = 6)
Input resistance	1.1 ± 0.2 GΩ (n = 8)	1.4 ± 0.2 GΩ (n = 9)
τ	11.8 ± 1.4 ms (n = 7)	12.2 ± 1.0 ms (n = 9)
